# Cachexia-Affected Survival Based on Inflammatory Parameters Compared to Complex Conventional Nutritional Assessments in Patients with Pancreatic Cancer and Other Gastrointestinal Tumors—The CONKO 020 Investigation

**DOI:** 10.3390/cancers16061194

**Published:** 2024-03-18

**Authors:** Johanna W. Meyer-Knees, Janina Falkenthal, Dominik Geisel, Christopher C. M. Neumann, Georg Hilfenhaus, Lars U. Stephan, Wenzel Schöning, Thomas Malinka, Johann Pratschke, Sebastian Stintzing, Uwe Pelzer

**Affiliations:** 1 Division of Oncology and Hematology, Berlin Institute of Health, Charite Campus Virchow Klinikum, Freie Universität Berlin, Humboldt Universität zu Berlin, 13353 Berlin, Germany; johanna.meyer-knees@charite.de; 2 KONZ-E-B-T CARE GmbH Zentrum für Ernährung, Beatmung und Therapie, 13435 Berlin, Germany; j.falkenthal@konzebt-care.de; 3 Division of Radiology, Berlin Institute of Health, Charite Campus Virchow Klinikum, Freie Universität Berlin, Humboldt Universität zu Berlin, 13353 Berlin, Germany; dominik.geisel@charite.de; 4 Division of Oncology and Hematology, Berlin Institute of Health, Charite Campus Mitte, Freie Universität Berlin, Humboldt Universität zu Berlin, 10117 Berlin, Germany; christopher.neumann@charite.de (C.C.M.N.); georg.hilfenhaus@charite.de (G.H.); lars.stephan@charite.de (L.U.S.); sebastian.stintzing@charite.de (S.S.); 5 Division of Surgery, Berlin Institute of Health, Charite Campus Mitte-Campus Virchow Klinikum, Freie Universität Berlin, Humboldt Universität zu Berlin, 13353 Berlin, Germany; wenzel.schoening@charite.de (W.S.); thomas.malinka@charite.de (T.M.); johann.pratschke@charite.de (J.P.)

**Keywords:** cachexia, PDAC, survival, IL-6, CRP, systemic inflammation

## Abstract

**Simple Summary:**

Pancreatic adenocarcinoma is a disease with multifactorial interactions that still has a devastating prognosis. Cachexia symptoms frequently impair patient survival. We commonly diagnose this later in the course of the disease once clinical signs become more evident. Early diagnosis is often difficult using conventional measurements. In the course of the disease, its discrimination from cancer progression is challenging and often overlaps. The aim of this study was to investigate whether conventional nutritional assessments or suitable laboratory parameters better predict the prognosis for cachexia-affected survival. We used established conventional methods, such as clinical and electrophysiological assessments, and compared them with inflammatory laboratory parameters. In addition, we used a control group with other gastrointestinal tumors to differentiate marker specificity. In patients with pancreatic adenocarcinoma, we were able to show that inflammatory parameters as well as albumin can determine the prognosis earlier and more specifically than conventional methods. From this, simpler diagnostic criteria could be derived, which could possibly bring earlier intervention into discussion for subgroups.

**Abstract:**

Background: Pancreatic adenocarcinoma (PDAC) is still a complex, devastating disease. Cachexia symptoms frequently impair patient survival. This accompanying syndrome is commonly diagnosed late, when clinical signs become evident. Early diagnosis using conventional measurement methods is often difficult, and the discrimination of this disease from cancer progression is challenging and often overlaps. The aim of this study was to analyze whether conventional nutritional assessments or laboratory biomarkers are better predictive tools for the early detection of patients at risk of reduced survival. Methods: We analyzed a prospective predefined cohort of 182 patients with gastrointestinal cancer, 120 patients with PDAC and—as controls—62 patients with other gastrointestinal adenocarcinoma (oAC), from whom we have sufficient data of protocol-defined conventional nutritional assessments, clinical data, and specific laboratory parameters. Results: at the time of tumor diagnosis, high inflammatory biomarkers (c-reactive protein (CRP), interleukin-6 (IL-6)) and albumin serum levels were associated with impaired OS in PDAC patients, but not in patients with oAC. Hemoglobin, body mass index (BMI), and bioelectrical assessments alone did not have a prognostic impact at the time of diagnosis. In a multivariate analysis, only CRP (HR 1.91 (1.25–2.92), *p* = 0.003) was found to be an independent prognostic factor in PDAC patients. Over the course of the disease in PDAC patients, inflammatory biomarkers, albumin, hemoglobin, and bioelectrical assessments were associated with impaired OS. In multivariate testing, CRP (HR 2.21 (1.38–3.55), *p* < 0.001) and albumin (HR 1.71 (1.05–2.77), *p* = 0.030) were found to be independent prognostic factors in PDAC patients. Conclusion: Specifically for PDAC patients, high inflammatory index and albumin serum levels potentially represent a sufficient early surrogate marker to detect patients at high risk of impaired OS better than complex conventional methods. These findings could help to identify patients who may benefit from early therapeutic interventions.

## 1. Introduction

Pancreatic ductal adenocarcinoma (PDAC) is one of the four leading causes of cancer death and is predicted to become the second most common cause by 2030 [1,2]. Despite the progress in medical research in recent decades, the overall 5-year survival rate is still nearly 10% [3]. Given the aggressive nature of cancer metabolism and the lack of effective screening tools, diagnosis most often occurs at a palliative cancer stage [4,5].

Progressive weight loss is often observed in PDAC patients [6]. Up to 20% of all cancer deaths worldwide are assumed to be caused by cancer cachexia, a complex and heterogeneous metabolic disorder [7,8]. Gastrointestinal malignancies have the highest incidence of cancer cachexia [9,10]. There is a strikingly high incidence of cachexia of up to 80 percent at time of diagnosis, and the most severe forms of cachexia are diagnosed in PDAC patients [11,12]. Cachexia is associated with poor patient outcomes since it causes increased toxicity of treatment, lower response rates, and worse performance status, having an adverse effect on quality of life and overall survival (OS) [8].

Despite its high prevalence and mortality rates, in clinical routines, cachexia is rarely diagnosed or treated [6]. Easily accessible and simple methods are needed to predict severe forms of cancer cachexia and to identify patients at risk for impaired OS who might benefit from early therapeutic interventions.

Inflammation plays a decisive role in the emergence of severe cancer cachexia [13,14]. The cancer secretes proinflammatory cytokines for self-preservation and enhanced growth. In response to cancer activity, the host immune system is activated, and acute phase proteins are secreted [15,16]. The systemic inflammation has peripheral and central effects leading to the degradation of muscle mass, adipose tissue, and anorexia [17,18,19].

With the increased understanding of the pivotal role of systemic inflammation in the onset and progression of cancer cachexia, a corresponding shift in how cancer cachexia is diagnosed and treated is needed. We assume that PDAC patients with high levels of systemic inflammation are more likely to develop severe forms of cancer cachexia and subsequently show an impaired mOS. With our study, we aimed to explore a more accurate prediction of cancer cachexia and inflammatory processes in PDAC patients. We assume that the prediction of OS by inflammatory parameters is more precise than by conventional cachexia parameters. We compared CRP, as a general marker of systemic inflammation, and IL-6, as a specific cytokine. We hypothesized that IL-6 would be a more sensitive marker than CRP in the prognostication of cancer cachexia. We analyzed the inflammatory biomarkers, CRP and IL-6, and other laboratory parameters (hemoglobin, albumin), as well as nutritional parameter body mass index (BMI) and bioelectrical assessments (extracellular mass/ body cell mass index (ECM/BCM index), phase angle), regarding their impact on OS at time of diagnosis. We assessed the impact of inflammation serum levels and nutritional indices over the course of disease. We compared the parameters’ impact on patients with PDAC and other gastrointestinal adenocarcinomas (oAC) as controls.

## 2. Materials and Methods

### 2.1. Study Cohort

From February 2013 to July 2018, we prospectively enrolled 198 patients at our outpatient center: Charité Cancer Center. Seven subjects with confirmed PDAC diagnosis and nine patients with oAC diagnoses were screened out due to insufficient clinical follow-up data or additional underlying other cancer type. In total, 182 patients had sufficient data for the predefined analysis: 120 PDAC patients and 62 patients with oAC (biliary tract *n* = 43, colorectal *n* = 18, esophageal *n* = 1). Inclusion criteria were a pathologically confirmed PDAC or oAC diagnosis, an inoperable stage of disease (stage III or IV), age of 18 years or older, outpatient treatment setting and willingness to undergo additional nutritional assessments and questionnaire. All patients consented to anonymous data processing. Ethics approval was obtained (EA1/168/23).

### 2.2. Data Collection and Study Definitions

The patients were followed up over the course of the disease, and the required parameters were assessed monthly or at shorter intervals, as indicated by the clinician or dietitian. Blood tests were taken on the same day as the clinician and nutritional work up. A certified nutritionist undertook the detailed dietary survey and carried out the bioelectrical impedance analysis (BIA) measurement with the determination of the ECM/BCM index and phase angle. BIA is an accredited method for assessing body composition. It uses the electric characteristics of the human body to measure the BIA variables. An alternating electric field is generated via skin electrodes. The ohmic resistance of the total body water (resistance) and the capacitive resistance created by cell membranes (reactance) are measured. Malnutrition, which is associated with a loss of body cells, is associated with lower reactance. Reactance results in a phase shift between current and voltage. This phase shift is expressed in degrees and is called the phase angle. The fat-free mass (FFM) of the body is composed of body cell mass (BCM) and extracellular mass (ECM). The BCM represents all metabolically active cells (organ cells and muscle tissue), while the ECM represents the non-cellular portion of the FFM. In good physical condition, BCM is higher than ECM. Elevated values may reflect malnutrition [20,21].

We defined OS as the duration from tissue pathology confirmation of cancer to death or the last confirmed contact. In the case of an R0/R1 cancer resection and later relapse, we defined the date of relapse to death or to the last confirmed contact as OS. The cut-off date of the survival analysis was set as 14 July 2020.

### 2.3. Statistical Analysis

We performed all analyses using IBM SPSS version 28 (IBM Corporation, Armonk, NY, USA). The results were considered statistically significant if *p* ≤ 0.05. Baseline parameters and patient characteristics are displayed as n and percent or median and interquartile range (IQR), as appropriate. Follow-up data were summarized as one mean value per patient. For survival analyses, we used a Kaplan–Meier estimation and log-rank as well as Cox regression testing. Significant parameters in univariate testing underwent further multivariate analysis. For each parameter, we split our cohort into two groups according to the median. Analyses were conducted for both baseline parameters and follow-up parameters, with the subsequent comparison of results.

## 3. Results

### 3.1. Clinical and Demographic Data in PDAC Patients Compared to Patients with oAC

Out of 198 patients, 182 patients, including 120 patients (65.9%) with PDAC and 62 patients (34.1%) with oAC (controls), were analyzed (Table 1). At the time of analysis, 107 (89.2%) patients with PDAC and 54 (87.1%) patients with oAC had died. The median OS in PDAC was 17.8 (11.9–22.9) months and 20.1 (10.3–35.8) months in oAC patients. The gender distribution and median age were similar in both groups. The median parameters at time of tumor diagnosis and over the course of disease are displayed in Table 1.

### 3.2. Effects of Parameters on Survival at the Time of Diagnosis

**Figure 1:**
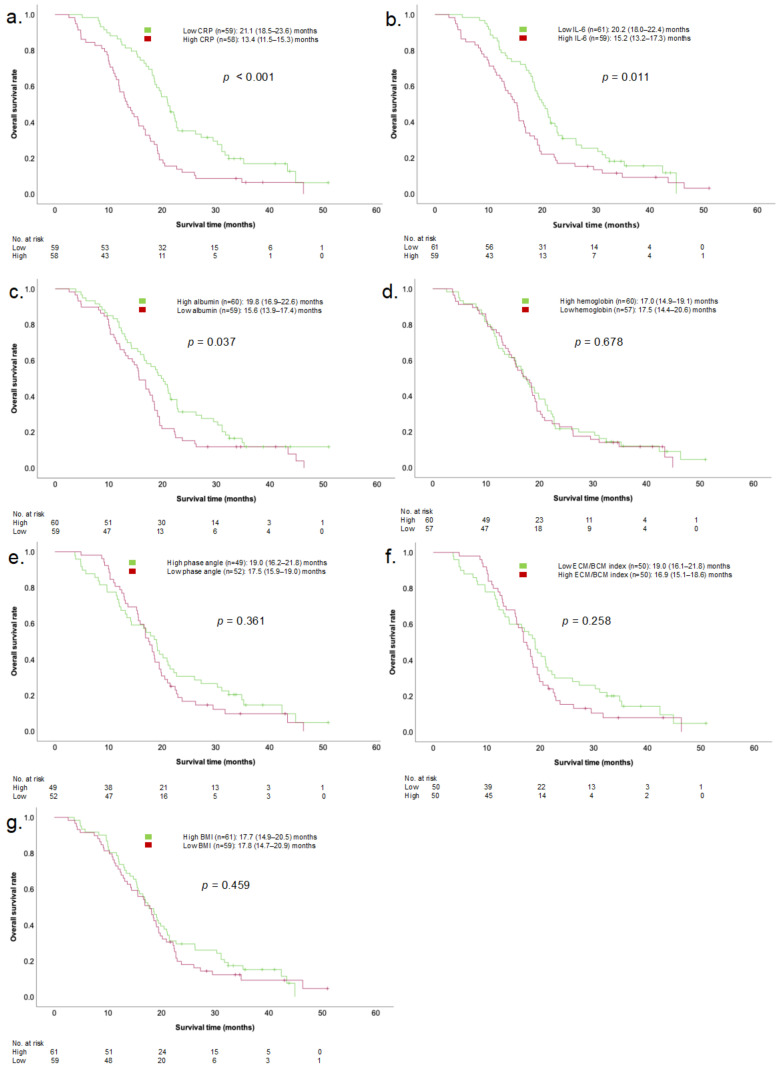
Kaplan–Meier univariate comparisons of mOS in PDAC patients based on CRP (**a**), IL-6 (**b**), albumin (**c**), hemoglobin (**d**), phase angle (**e**), ECM/BCM index (**f**), and BMI (**g**) at time of diagnosis. mOS, median overall survival; PDAC, pancreatic ductal adenocarcinoma; CRP, c-reactive protein; IL-6, interleukin 6; ECM/BCM, extracellular mass/body cell mass, BMI; body mass index.

PDAC patients with high CRP and IL-6 serum levels at the time of diagnosis showed significantly impaired OS compared to patients with low inflammatory serum levels. The mOS in patients with low CRP serum levels (*n* = 59) was 21.1 (18.5–23.6) months, and in patients with high CRP serum levels (*n* = 58), it was 13.4 (11.5–15.3) months (*p* < 0.001). Patients with low IL-6 serum levels (*n* = 61) had an mOS of 20.2 (18.0–22.4) months and patients with high IL-6 serum levels (*n* = 59) had an mOS of 15.2 (13.2–17.3) months (*p* = 0.011). Patients with low serum albumin levels (*n* = 59) showed a significantly worse OS (low albumin (*n* = 59): 15.6 (13.9–17.4) months vs. high albumin (*n* = 60): 19.8 (16.9–22.6) months, *p* = 0.037). In PDAC patients, at the time of initial tumor diagnosis, BMI and BIA parameters as well as hemoglobin did not have prognostic impact on mOS (Figure 1).

Table 2 shows the univariate and multivariate Cox regression analyses for PDAC patients. In the univariate model, serum levels of CRP (HR 2.17 (1.47–3.22), *p* < 0.001) and IL-6 (HR 1.63 (1.12–2.41), *p* = 0.012), as well as albumin (HR 1.50 (1.02–2.21), *p* = 0.039), were associated with impaired mOS. In multivariate testing, only CRP was shown to be an independent prognostic factor of survival (HR 1.91 (1.25–2.92), *p* = 0.003).

In patients with oAC, neither inflammatory parameters nor other conventional cachexia parameters were shown to be associated with OS at the time of diagnosis.

### 3.3. Effects of Parameters on Survival over the Course of the Disease

**Figure 2:**
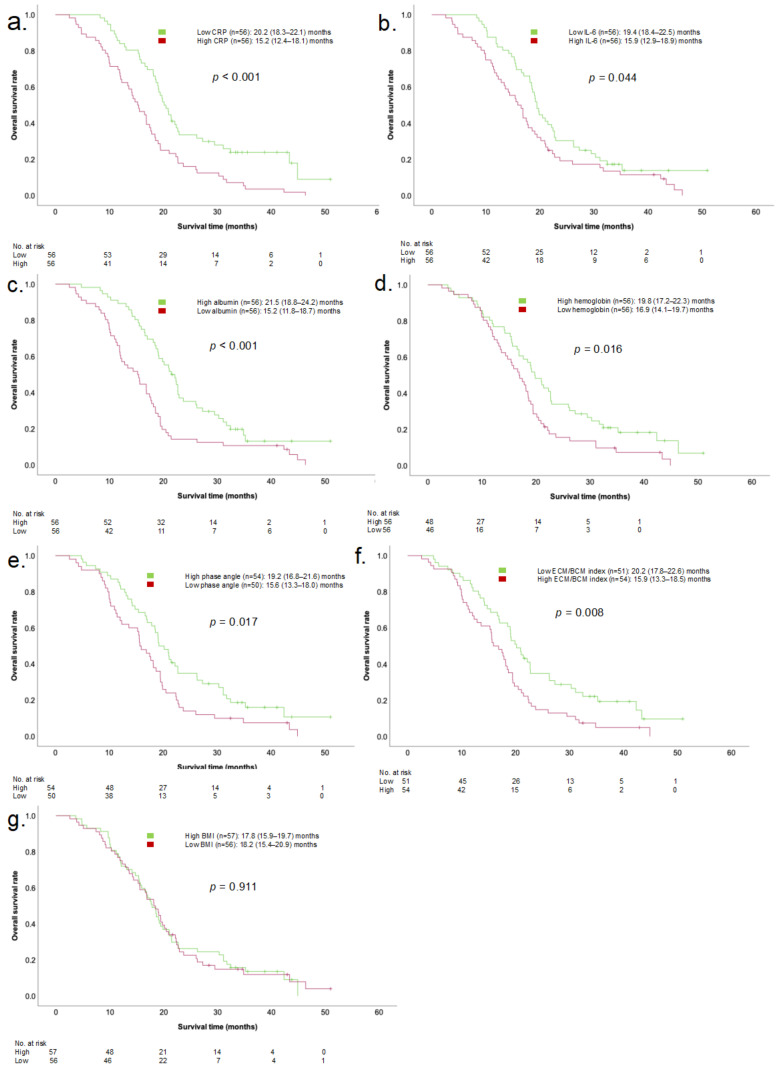
Kaplan–Meier univariate comparisons of mOS in PDAC patients based on the follow-up parameters of CRP (**a**), IL-6 (**b**), albumin (**c**), hemoglobin (**d**), phase angle (**e**), ECM/BCM index (**f**), and BMI (**g**). mOS, median overall survival; PDAC, pancreatic ductal adenocarcinoma; CRP, c-reactive protein; IL-6, interleukin 6; ECM/BCM, extracellular mass/body cell mass; BMI; body mass index.

Figure 2 shows the Kaplan–Meier analysis of the follow-up parameters of PDAC patients. Over the course of disease, PDAC patients with high CRP (*n* = 56) and IL-6 (*n* = 56) serum levels showed an impaired OS of 15.2 (12.4–18.1) months (*p* < 0.001) and 15.9 (12.9–18.9) months (*p* = 0.044), respectively. Low albumin (*p* < 0.001) and hemoglobin (*p* = 0.016) serum levels were associated with impaired OS in PDAC patients. BIA parameters showed a prognostic impact on OS. Patients with high ECM/BCM index (*p* = 0.008) and low phase angle (*p* = 0.017) showed an impaired OS. BMI did not show a significant association with survival. In multivariate Cox regression testing (Table 3), only CRP (HR 2.21 (1.38–3.55), *p* < 0.001) and albumin (HR 1.71 (1.05–2.77), *p* = 0.030) serum levels were shown to be independent prognostic parameters.

In patients with oAC, only for low albumin serum levels was a negative prognostic impact on mOS demonstrated (HR 1.83 (1.03–3.27), *p* = 0.041).

## 4. Discussion

In addition to advances in anticancer strategies, improving supportive therapy for patients with aggressive tumor diseases, such as pancreatic cancer, is crucial for patient outcomes. Cancer cachexia is one of the most common tumor-associated complications, impairing patients’ quality of life and prognosis. Diagnostic parameters are needed to identify PDAC patients with a high inflammatory status associated with cancer cachexia and poor survival.

To date, little is known about prognostic biomarkers of cancer cachexia. The understanding of cachexia as an inflammation-related metabolic dysregulation opens up new possibilities in the early detection of cachexia [13,14]. Despite several investigations about the correlation of inflammatory parameters and cachexia, early biomarkers and therapeutic intervention strategies are still under discussion [22,23].

We found that in PDAC patients, but not in patients with oAC, high inflammatory biomarker (CRP and IL-6) serum levels at time of initial tumor diagnosis have a negative impact on OS (Figure 1, Table 2). Among the other cachexia parameters, only albumin showed a negative prognostic impact on OS. In multivariate analysis, CRP remained an independent prognostic parameter for impaired OS. Over the course of the disease, inflammatory biomarker serum levels (CRP, IL-6) were shown to remain prognostic. Additionally, BIA parameters and albumin, as well as hemoglobin levels, proved to be valid markers to identify patients at risk of impaired OS. BMI, commonly used in routine clinical practice to evaluate patients’ nutritional status, did not show a statistically significant association with survival. In multivariate testing, only CRP and albumin serum levels remained independent prognostic parameters of survival. In patients with oAC—serving as controls—none of the parameters at the time of diagnosis had a prognostic impact. Over the course of the disease, only albumin serum levels were associated with impaired OS. The heterogeneous results observed in PDAC patients and patients with oAC indicate a specific character of the systemic, metabolic cancer burden present in PDAC patients. Based on our analysis, we cannot confirm that the results can be generalized to other tumor entities. Further studies are needed to investigate the significance of inflammatory parameters in the prognostication of other cancer types.

In the past, cancer cachexia was commonly understood as simple malnutrition, but the growing recognition of cachexia as an inflammation-driven metabolic disorder has shifted this perspective [24]. Recently, inflammation has been increasingly discussed as a relevant prognostic factor in cancer patients. Inflammatory serum markers have been correlated with OS, risk of recurrence, and treatment response [25,26]. The modified Glascow Prognostic Score (mGPS) combines CRP and albumin serum levels [27]. It reflects patients’ inflammatory and nutritional statuses. Previous studies and meta-analyses have described the prognostic role of the mGPS in various cancer entities [28,29]. It was correlated with poor OS irrespective of the disease stage and treatment [28]. This supports our finding of CRP and albumin as early prognostic parameters at the time of cancer diagnosis. It remains controversial whether elevated CRP serum levels could be an expression of disease progression associated with impaired OS. The results of another study showing a correlation between CRP levels and OS after stratification by disease extent contradict an increase in CRP only in the context of tumor progression [7]. In various tumor entities, an association of elevated IL-6 serum levels with weight loss as well as poor performance status was shown [30,31,32]. It has been demonstrated that IL-6 is overexpressed in pancreatic tissue and that pancreatic cancer patients with cachexia have higher serum levels than patients without it [33]. In our analysis, IL-6 was shown to only have a prognostic impact in univariate testing. Thus, using our analysis, we could not prove IL-6 to be a more sensitive parameter than the acute-phase protein CRP. An explanation for this might be the multifactorial nature of cancer cachexia. All involved cytokines lead to the synthesis of acute-phase proteins. Further studies with a larger sample size and a prospective study design are needed to validate the prognostic role of IL-6 in the context of cancer cachexia in PDAC patients.

In previous studies, it has been shown that a decline in hemoglobin levels is linked to decreased muscle mass, elevated mortality rates, and ultimately, a poorer overall prognosis [34]. Our findings align with this understanding, as demonstrated by Kaplan–Meier analyses, indicating that pancreatic cancer patients with low hemoglobin levels experience impaired OS throughout their disease course. Notably, in the early stages of the disease, hemoglobin levels did not show prognostic significance in our analysis.

Recent research has shown that cancer cachexia cannot be considered as simple weight loss. It was demonstrated that the conventional nutritional therapy of cancer patients alone does not improve disease- or treatment-related morbidity or mortality [24]. Thus, the increased mortality in cancer cachexia cannot be exclusively attributed to the effects of malnutrition. This supports our findings indicating that BMI has no significant association with mOS.

Regarding BIA parameters, various studies have demonstrated their prognostic significance for OS in patients with different cancer types [35,36]. These findings support our results on phase angle and ECM/BCM follow-up parameters. At the initial cancer diagnosis, the BIA parameters in our study did not have a significant impact on OS. We attribute this to the progressive deterioration of body composition throughout the disease course, which is subsequently reflected in abnormal BIA parameters.

Tumor cachexia represents a highly significant challenge in routine clinical practice. Despite our current understanding, the management of affected patients has yet not been notably improved. This could be attributed to the recognition of cancer cachexia extending beyond nutritional considerations and representing a rather metabolic phenomenon driven by systemic inflammatory responses. To the best of our knowledge, our study is the first to compare inflammatory biomarkers, other laboratory parameters, and clinical and device-based nutritional assessments. We create a holistic view of the challenge of cancer cachexia and do not simply focus on one dimension of diagnostics.

The limitations of our study are the small sample size and the retrospective study design. Due to the natural course of the disease and frequency of individual presentation, we had dropouts resulting in incomplete follow-up data. Previous drug therapies or surgeries, as well as the presence of metastases, infections or nutritional therapies were not assessed. These factors may affect serum levels of biomarkers or device-based tests. These factors should be included in future clinical trials on prognostic factors of cancer cachexia. In our analysis, we compared CRP, as a general marker of systemic inflammation, and IL-6, as a specific factor in emergence of cancer cachexia, as two biomarkers that would be easily accessible in routine clinical practice. In future clinical trials, it would be preferable to create a prognostic parameter profile (including additional inflammatory markers). Cytokine profiling should be performed to identify additional inflammatory markers and explore the pathways by which inflammatory markers contribute to the development of cachexia to understand the complex pathological process and to subsequently identify effective treatment strategies.

## 5. Conclusions

In summary, inflammatory biomarkers in combination with albumin compared to other conventional nutritional parameters may represent earlier and more sensitive parameters in the prognostication of pancreatic cancer. The parameters could serve as early indicators to identify patients at risk of severe forms of cancer cachexia and subsequently impaired OS, even in the absence of clinically visible signs of malnutrition. We can assume that the extent of systemic, metabolic cancer burden shows a specific characteristic and is reflected early by inflammatory parameters in PDAC patients but not in patients with oAC. Identifying patients with high inflammatory baseline parameters and consecutively poor prognosis is crucial for designing intervention research in the field of inflammation-targeted cachexia treatments. The identified high-risk patients may profit from early, targeted and individualized nutritional intervention.

## Figures and Tables

**Table 1:** Patient characteristics and parameters at time of diagnosis and over the course of the disease. Categorical variables are shown as n (%), and continuous variables are shown as median (IQR).

Characteristics	PDAC		oAC	
Male sex				
n (%)	68 (56.7)		34 (54.8%)	
Age (years)				
Median (IQR)	63.9 (55.2–71.7)		63.0 (54.8–70.2)	
OS			20.1 (10.3–35.8)	
Median (IQR)	17.8 (11.9–22.9)			
Deceased				
n (%)	107 (89.2)		54 (87.1)	
Parameter	Baseline	Follow-up	Baseline	Follow-up
CRP (mg/L)				
Median (IQR)	4.3 (1.7–15.6)	12.9 (6.6–29.4)	8.8 (4.5–20.9)	15.5 (7.8–34.5)
Missing	3	8	4	8
IL-6 (pg/mL)				
Median (IQR)	7.6 (4.3–12.0)	14.8 (8.9–25.5)	9.6 (5.6–20.4)	15.3 (8.6–24.1)
Missing		8	1	7
Albumin (g/dL)				
Median (IQR)	38.3 (35.2–40.9)	36.7 (24.2–38.9)	37.9 (34.0–40.2)	37.3 (33.9–39.8)
Missing	1	8	6	7
Hemoglobin (g/dL)				
Median (IQR)	11.5 (10.5–12.6)	10.8 (10.0–11.8)	11.6 (10.6–12.3)	10.8 (9.8–11.7)
Missing	3	8	0	6
Phase angle (°)				
Median (IQR)	4.4 (3.7–5.0)	4.0 (3.7–4.5)	4.7 (4.0–5.3)	4.6 (4.0–5.0)
Missing	19	16	8	10
ECM/BCM index				
Median (IQR)	1.3 (1.1–1.7)	1.5 (1.3–1.7)	1.2 (1.1–1.5)	1.3 (1.1–1.5)
Missing	20	15	9	11
BMI (kg/m^2^)				
Median (IQR)	22.9 (19.7–25.1)	22.5 (20.0–24.7)	22.8 (20.6–25.5)	22.8 (20.9–25.8)
Missing		7		7

OS; overall survival; PDAC, pancreatic ductal adenocarcinoma; CRP, c-reactive protein; IL-6, interleukin 6; ECM/BCM, extracellular mass/body cell mass; BMI; body mass index.

**Table 2:** Univariate and multivariate Cox regression analysis for PDAC patients with parameters at time of diagnosis showing the hazard ratios (HR) for an event (death) and the 95% confidence intervals (CI).

Parameter	Univariate Analysis			Multivariate Analysis		
	HR	95% CI	*p*	HR	95% CI	*p*
CRP > 4.3 mg/L	2.17	1.47–3.22	<0.001	1.91	1.25–2.92	0.003
IL-6 > 7.6 pg/mL	1.63	1.12–2.41	0.012	1.21	0.77–1.89	0.409
Albumin < 38.3 g/dL	1.50	1.02–2.21	0.039	1.015	0.74–1.77	0.539
Hemoglobin < 11.5 g/dL	1.08	0.74–1.59	0.679			
Phase angle < 4.4°	1.21	0.80–1.84	0.361			
ECM/BCM-Index > 1.3	1.27	0.84–1.94	0.259			
BMI < 22.9 kg/m^2^	1.16	0.79–1.70	0.460			

PDAC, pancreatic ductal adenocarcinoma; CRP, c-reactive protein; IL-6, interleukin 6; ECM/BCM, extracellular mass/body cell mass, BMI; body mass index.

**Table 3:** Univariate and multivariate Cox regression analysis for PDAC patients with parameters over the course of disease, showing the hazard ratios (HR) for an event (death) and the 95% confidence intervals (CI).

Parameter	Univariate Analysis			Multivariate Analysis		
	HR	95% CI	*p*	HR	95% CI	*p*
CRP > 12.9 mg/L	2.08	1.40–3.10	<0.001	2.21	1.38–3.55	<0.001
IL-6 > 14.8 pg/mL	1.50	1.01–2.22	0.046	0.71	0.43–1.16	0.170
Albumin < 36.7 g/dL	2.00	1.33–2.97	<0.001	1.71	1.05–2.77	0.030
Hemoglobin < 11.5 g/dL	1.63	1.09–2.43	0.017	1.21	0.76–1.93	0.429
Phase angle < 4.0°	1.64	1.09–2.47	0.018	1.41	0.75–2.64	0.283
ECM/BCM Index > 1.5	1.74	1.15–2.62	0.008	1.12	0.59–2.12	0.731
BMI < 22.5 kg/m^2^	1.02	0.69–1.51	0.911			

PDAC, pancreatic ductal adenocarcinoma; CRP, c-reactive protein; IL-6, interleukin 6; BMI; body mass index; ECM/BCM, extracellular mass/body cell mass.

## Data Availability

The database is stored on the Charite—Universitätsmedizin Berlin own server in a legally secure manner. All data has been saved and checked in pseudonymised form for analysis. The pseudonymised data set can be requested from the project manager of the investi-gation (UP), if there is contractual legal protection.
